# Editorial: Progressive fibrosing interstitial lung disease: from bench to bedside

**DOI:** 10.3389/fmed.2023.1277909

**Published:** 2023-09-01

**Authors:** Julien Guiot, Makon-Sébastien Njock

**Affiliations:** ^1^Department of Respiratory Medicine, University Hospital of Liège, Liège, Belgium; ^2^Laboratory of Pneumology, GIGA Research Center, University of Liège, Liège, Belgium

**Keywords:** progressive fibrosing interstitial lung disease, fibrotic lung diseases, biomarkers, diagnosis, prognosis, predictive model

## Introduction

Pulmonary fibrosis is a debilitating and potentially progressive lung disease characterized by the excessive accumulation of fibrotic tissue within the lung parenchyma, leading to impaired gas exchange and respiratory failure. Despite significant advances in our understanding of the pathogenesis and management of pulmonary fibrosis, the disease remains a therapeutic challenge. To date, the gold standard for the diagnosis of progressive pulmonary fibrosis (PPF) is a holistic evaluation through multimodal assessment including the analysis of clinical symptoms, pulmonary function test (PFT), histopathological evaluation and high-resolution computed tomography (HRCT) of the chest (1).

This editorial aims to shed light on the current state of research and clinical approaches in the field of PPF, emphasizing the need of early biomarkers helping clinicians to identify PPF at the earliest stage and also new potential therapeutic targets aiming to stop the uncontrolled fibrotic process. This Research Topic currently includes 15 original research articles on the diagnosis/prognosis of PPF, based on the identification of news biomarkers, multi-scale analysis of clinical informations and parameters, which results on the development of prediction models. These research articles are from interdisciplinary collaboration (clinicians, researchers and industry partners) and emerging technologies. All contributions to this Research Topic focus on one or more of the research areas highlighted above.

## Clinical management

Despite the recent progress in understanding the molecular mechanisms of pulmonary fibrosis, the clinical management of patients with progressive disease remains a complex challenge. Indeed, as stated in the recent guidelines (2), clinicians have to wait the reduction of PFT and/or a PPF based on imaging analysis. ATS/ERS recommendations state that diagnostic criteria for PPF are a combination of two criteria including: worsening symptoms over time, HRCT-proven fibrotic progression and/or an absolute decline from baseline in FVC (≥5%) or DLCO (≥10%) over 1 year of follow-up.

In a review article, Stanel and Rivera-Ortega have discussed about the perspectives in early diagnosis and monitoring for progressive fibrosing interstitial lung diseases (PF-ILD). In a retrospective study, Chiu et al. have examined the prognostic relevance of PPF definitions, and shown that it did not differ between simplified PPF, INBUILD and ATS/ERS/JRS/ALAT 2022 criteria. A recent study by Takei et al. highlights the need to consider an evaluation of health-related quality of life when assessing PPF in patients with idiopathic pulmonary fibrosis.

Currently available therapies, such as antifibrotic drugs, primarily target the fibrotic process but have limited efficacy in preventing early disease progression. As a result, a comprehensive and personalized approach to patient care is required. Clinical trials evaluating combination therapies, precision medicine approaches, and novel drug delivery systems hold promise for improving patient outcomes and quality of life (Wu et al.).

## Emerging technologies and biomarkers

The advent of advanced technologies has revolutionized the field of pulmonary fibrosis research. High-throughput genomics, proteomics, and metabolomics have allowed researchers to identify novel biomarkers associated with disease progression and prognosis. These biomarkers not only aid in early diagnosis but also serve as valuable tools for monitoring treatment response and predicting outcomes. Additionally, innovative imaging techniques, such as HRCT and functional lung imaging, contribute to a more precise assessment of disease severity and progression (3).

With this topic, several studies have developed predictive/prognostic model for PF-ILD based on machine learning algorithms by combining clinical informations (Shao et al.; Lee et al.; Zhang et al.; Niu et al.).

## Translational research

Translational research acts as the critical link between bench research and clinical practice, facilitating the transition of promising experimental findings into real-world applications. In the context of PPF, translational studies have demonstrated the efficacy of several novel therapeutic interventions in preclinical models (4). From anti-fibrotic drugs to gene and cell-based therapies, these advancements offer hope for the development of effective treatments that can halt or even reverse disease progression.

### Bridging the gap between bench and bedside

The journey from bench to bedside is essential in translating scientific discoveries into effective clinical interventions. Bench research plays a pivotal role in unraveling the underlying molecular mechanisms driving PPF. Investigating key cellular pathways, such as epithelial-mesenchymal transition (EMT), myofibroblast activation, and dysregulated immune responses, has contributed significantly to our understanding of disease pathogenesis (5–7). Fundamental discoveries led to the identification of potential therapeutic targets, paving the way for novel treatment strategies.

## Collaboration and data sharing

To accelerate progress in the field of PPF, collaboration and data sharing among researchers, clinicians, and industry partners are of paramount importance. Sharing research findings, clinical data, and biological samples through established networks and databases can foster synergistic efforts and enable more comprehensive analyses (Tomassetti et al.; Quan et al.). Moreover, collaborative efforts facilitate the validation of preclinical research findings in diverse patient populations, ultimately leading to more robust and reliable clinical recommendations.

## Importance of patient advocacy and support

Lastly, it is crucial to acknowledge the invaluable role of patient advocacy groups and support networks in raising awareness about PPF. These organizations provide a platform for patients, caregivers, and researchers to collaborate, share experiences, and advocate for improved access to care, research funding, and better overall understanding of the disease. By amplifying patient voices and perspectives, we can drive meaningful change in the field and ensure that scientific advancements are translated into tangible benefits for those affected by the disease.

## Conclusion

The journey from bench to bedside in the realm of PPF research holds immense potential for advancing our understanding and treatment of this debilitating disease of high unmet medical need. Through the integration of bench discoveries, translational research, and innovative clinical approaches, we can bridge the gap between scientific knowledge and patient care. By fostering collaboration, embracing emerging technologies, and prioritizing patient advocacy, we can work toward a future where PPF is no longer an insurmountable challenge, but a conquerable condition.

## Author contributions

JG: Writing—original draft, Writing—review and editing. M-SN: Writing—original draft, Writing—review and editing.
